# Does hallux valgus impair physical function?

**DOI:** 10.1186/s12891-018-2100-0

**Published:** 2018-05-29

**Authors:** Akinobu Nishimura, Naoya Ito, Shigeto Nakazora, Ko Kato, Toru Ogura, Akihiro Sudo

**Affiliations:** 1 Departments of Orthopaedic Surgery, Mie University Graduate School of Medicine, 2-174 Edobashi, Tsu City, Mie 514-8507 Japan; 2 Departments of Orthopaedic and Sports Medicine, Mie University Graduate School of Medicine, Tsu City, 514-8507 Mie Japan; 3 Department of Orthopaedic Surgery, Suzuka Kaisei Hospital, Suzuka City, 513-8505 Mie Japan; 4 Clinical Research Support Center, Mie University Hospital, 2-174 Edobashi, Tsu City, Mie 514-8507 Japan

**Keywords:** Hallux valgus, Physical function, Knee osteoarthritis, Epidemiology elderly

## Abstract

**Background:**

The relationships between radiographic hallux valgus (HV) and various physical functions independent of knee osteoarthritis (KOA) were examined among residents of a mountain village in Japan.

**Methods:**

Study participants were recruited from mountain village residents aged ≥50 years. Participants’ height, weight, and body mass index (BMI) were measured, and baseline data, including age, sex, and foot pain, were obtained using interviews and questionnaires. Radiography of the feet and knees was performed to assess the presence of HV (HV angle ≥20°) and KOA (Kellgren-Lawrence grade ≥ II). Grip strength, 6-m walk at usual and maximum speeds, single-leg stance time, and stand up from a chair time were evaluated as physical function performance tests. Plantar pressure patterns were also examined.

**Results:**

Moderate-severe HV (HV angle ≥30 degrees), impaired grip strength and maximum walking speed, and painful HV reduced usual and maximum walking speeds independent of KOA. Hallux plantar pressure decreased according to the HV angle. Hallux plantar pressure was significantly lower in painful HV than in the no HV feet or painless HV.

**Conclusions:**

Moderate-severe HV deformity and HV-related pain impaired physical function independent of KOA. By controlling the pain and severe deformity of HV by treatments such as surgery, the physical function of HV patients might be improved.

## Background

Hallux valgus (HV) is one of the most common foot deformities in adults; it is characterized by abnormal angulation, rotation, and lateral deviation of the hallux at the first metatarsophalangeal joint [1]. The prevalence of HV has been reported as 58% in adult women and 25% in adult men (HV angle ≥15°) in the USA [2], 28.4% in adults (self-reported hallux valgus) in the UK [3], 64.7% (HV angle ≥15°) in a Korean population aged between 40 and 69 years [4], and 29.8% (HV angle > 20°) in a Japanese population aged over 65 years [5]. While HV is basically regarded as a structural deformity, there is debate surrounding the association between abnormal foot structure and related disability. Several studies have found no association between HV and disability, such as the Timed Up & Go test [6], walking speed [6–8], and balance tests [8]. On the other hand, increasing HV severity has been shown to negatively impact health-related quality of life and self-reported function [4, 9, 10], and HV has been linked to balance function [11] and increased fall risk in older adults [12, 13].

Our cohort study started in 1997 to investigate the epidemiology of knee osteoarthritis (KOA) [14, 15] and osteoporosis [16]. The study of HV started from 2009, and we reported the prevalence and risk factors for HV [5]. In our study [5], HV showed a significant relationship with KOA. In general, KOA is associated with lower physical extremity function [17]. However, no report has shown a relationship between HV and physical function after taking into account the existence of KOA.

The purpose of this cross-sectional study was to investigate whether HV affects physical function after taking into account KOA among elderly persons.

## Methods

Individuals aged ≥50 years were recruited from among the inhabitants of a mountain village in Japan. The main industry is the forest industry. The study was started in 1997, with follow-up every two years. All studies were held at the local hospital. This present study analyzed data from the seventh to tenth biennial examinations in 2009, 2011, 2013, and 2015, respectively. The number of participants in 2009, 2011, 2013, and 2015 was 314, 221, 223, and 204, respectively. For individuals who participated in two or more iterations of these four examinations, only the data from the earliest examination were included. For example, if an individual participated in 2009, 2011 and 2015, the data in 2009 were included in this study. Exclusion criteria were rheumatoid arthritis and past history of surgery for hallux valgus. A total of 562 participants (194 male, 368 female) were part of this study.

Before direct examination, a baseline questionnaire including name, age, sex, medical history, and pain was sent to the participants. Direct examination consisted of physical measurement, medical interview, physical examination, X-rays, blood tests, and physical function tests. We measured participants’ height and body weight, and the body mass index (BMI) was calculated as weight (kg) divided by height squared (m^2^). Medical interviews and physical examinations were performed one-on-one by orthopedic surgeons. Bunions were detected by inspection and palpation. Painful bunions were determined by applying pressure to the bunion. Knee X-rays were taken with the knee in a fully extended standing position. KOA was scored according to the Kellgren-Lawrence grading system [18]. Radiographic KOA was defined as grade ≥ 2. We took foot X-rays with participants standing upright with both feet on the cassette, as described by Saltzman [19]. The HV angle, which is formed by the bone axes of the first metatarsal and the first proximal phalanx, was consistently measured by the same examiner [20]. These imaging data were analyzed using Image J version 1.37 software (National Institutes of Health, Bethesda, MD, USA). Hallux valgus was deemed to be present if the HV angle was ≥20°, according to the Japanese Orthopaedic Society criteria. HV severity was classified as mild (≥20° and < 30°), moderate (≥30° and < 40°), or severe (≥40°). Participants reporting pain in the right or left hallux on most days of a month for at least 1 month in the previous year were classified as having right or left hallux pain. Participants with radiographic HV and self-reported hallux pain or a painful bunion checked by orthopedic surgeons were defined as having painful HV. If a participant had left hallux pain and right radiographic HV, that participant was not included in the painful HV group.

**Fig. 1:**
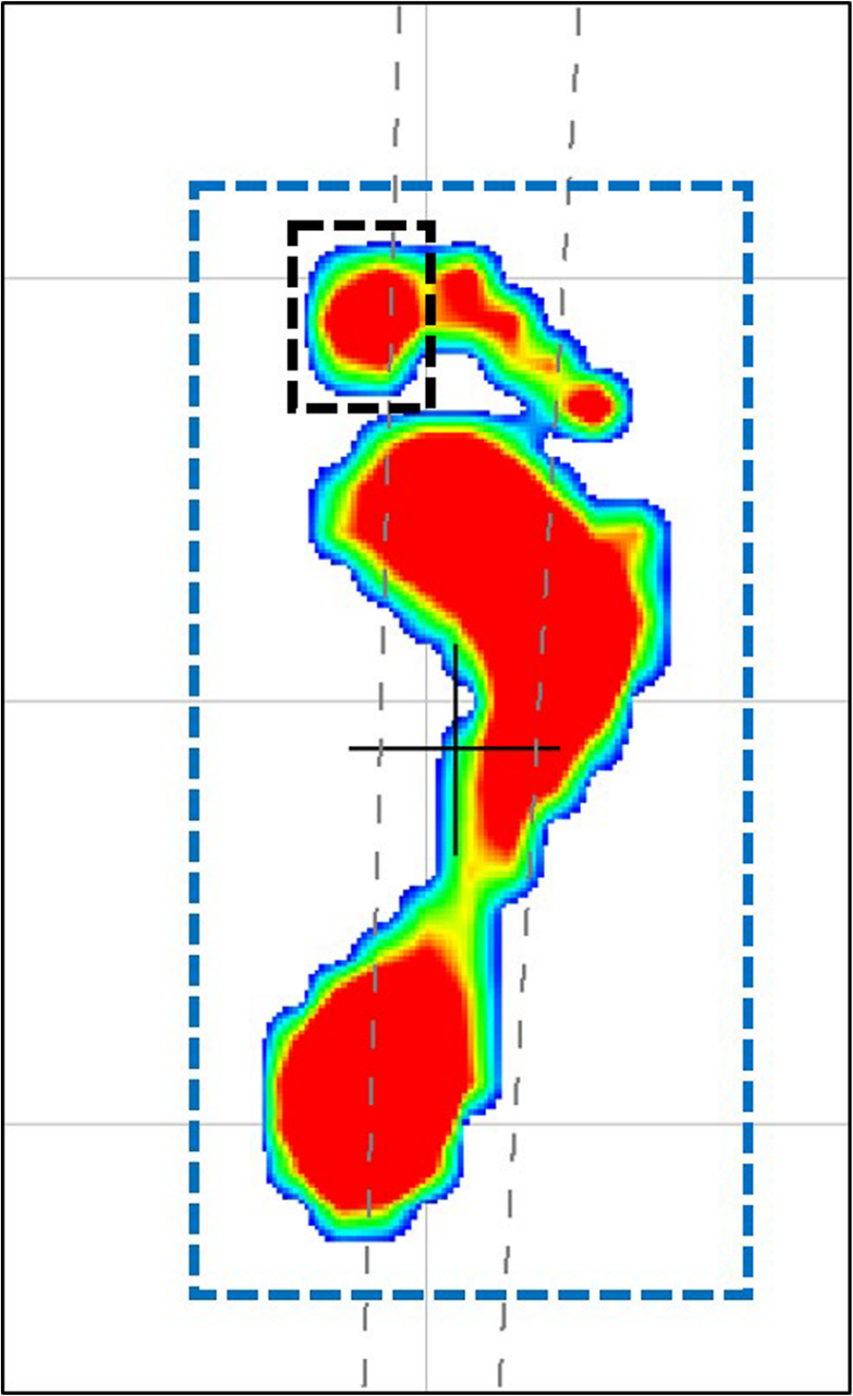
Representative footprint. Blue dotted line shows the region of interest (ROI) of a whole footprint, and the black dotted line shows the ROI of a hallux footprint for pressure analysis. The percentage of the hallux footprint is calculated as the hallux footprint pressure divided by the whole footprint

The physical function tests consisted of grip strength, 6-meter (m) walk at usual and maximum speeds, single-leg stance time with eyes open, and stand up from a chair time. Grip strength was measured using a Takei 5401 handgrip dynamometer (Takei Scientific Instruments Co., Niigata, Japan). The participants stood and held the dynamometer with the arm at right angles and the elbow by the side of the body. Two measurements were done for each hand. We used the average of the highest measurement of each hand. Participants were instructed to walk at their usual and maximum speeds. We measured the time was with a stopwatch. The data for plantar pressure patterns were taken using a gait analyzer (Walk Way MW 1000; Anima, Tokyo, Japan). This analyzer has a sheet length of 2.4 m, and this sheet was set in the middle of the 6-m walkway. In order to investigate how strongly participants pushed the ground with the great toe, the percentage of the hallux footprint in the whole footprint was examined. Each footprint was picked up using the gait analyzer (Fig. 1), and the hallux footprint and the whole footprint were enclosed, and the pressure was analyzed with analysis software (PREDAS MD-1000; Anima, Tokyo, Japan). The percentage of the hallux footprint was defined as [hallux footprint]/[whole footprint]. Examining the foot pressure patterns, the percentage of hallux footprint was checked, from which was calculated hallux pressure/whole foot pressure. We measured single-leg stance time with eyes open for both legs, and we calculated the average time. Participants were instructed to stand on one leg while elevating the contralateral limb. The time until the raised leg was touched on the floor was measured. The maximum time was 60 seconds (s). The stand up from a chair time was the time taken to stand from a chair five times with hands folded in front of the chest and feet flat on the floor.

Based on the worse HV angle in their feet, the participants were classified into a no HV group (HV angle < 20°) and an HV group (HV angle ≥20°). They were then classified into a no HV-mild HV group (HV angle < 30°) and a moderate-severe HV group (HV angle ≥30°). Finally, they were classified into a painful HV group and a no HV or painless HV group.

### Statistical analysis

Means ± standard deviations were calculated for variables unless otherwise noted. Associations among the physical characteristics between the groups were determined by the unpaired *t*-test or Fisher’s exact test. The relationships between physical functions (grip strength, single-leg stance time, standing up from a chair, and 6-m walking times at usual and maximum speeds) and HV (HV angle ≥20°), moderate-severe HV (HV angle ≥30°), or painful HV (HV angle ≥20° with pain) were evaluated by multiple linear regression analysis both after adjusting for age, sex, and BMI, and for age, sex, BMI, and KOA. The correlation between the HV angle and the percentage of the hallux footprint was analyzed by Pearson’s correlation coefficient. Percentages of the hallux footprint between no HV foot and HV, between no HV-mild HV and moderate-severe HV, and between no HV foot or painless HV and painful HV were analyzed by the Mann-Whitney test. The significance level was set at 5% in a two-tailed test. All data were analyzed using PASW Statistics for Windows version 22 (IBM, Armonk, NY, USA).

## Results

The overall prevalence of definite radiographic HV was 28.4% (160/562); it was 13.4% (26/194) in males and 36.4% (134/368) in females. The rates of mild, moderate, and severe HV in the 562 participants were 16.5% (93/562), 9.3% (52/562), and 2.7% (15/562), respectively.

Table 1 shows the physical characteristics of the participants who fulfilled the criteria. The HV group had 160 participants (26 male, 134 female), whereas the no HV group had 402 participants (168 male, 234 female). Participants in the HV group were significantly shorter and older than those in the no HV group, and the percentages of female and KOA were significantly higher in the HV group than in the no HV group. When participants were classified into a no HV-mild HV group and a moderate-severe HV group, participants in the moderate-severe HV group were significantly shorter and lighter than those in the no HV-mild HV group, and the percentages of female and KOA were significantly higher in the moderate-severe HV group than in the no HV-mild HV group. When participants were classified into a no HV or painless HV group and a painful HV group, the participants in the painful HV group were significantly shorter than those in the no HV or painless HV group, and the percentages of female and KOA were significantly higher in the painful HV group than in the no HV or painless HV group.

**Table 1:** Comparison of participants’ basic data between the no HV group and the HV group, between the no HV -mild HV group and the moderate-severe HV group, and between the no HV or painless HV group and the painful HV group

	No HV group	HV group	No HV-mild HV group	Moderate-severe HV group	No HV or painless HV group	Painful HV group
	(*n* = 402)	(*n* = 160)	(*n* = 495)	(*n* = 67)	(*n* = 529)	(*n* = 33)
Age (years)	**71.1 ± 8.8	73.3 ± 8.7	71.5 ± 8.9	73.4 ± 8.3	71.6 ± 8.8	74.0 ± 9.1
Sex (% female)	**58.2	83.8	**62.2	89.6	*64.1	87.9
Height (cm)	**154.6 ± 8.9	151.1 ± 7.8	**154.2 ± 8.7	149.7 ± 7.5	*153.9 ± 8.6	148.9 ± 9.0
Weight (kg)	**55.8 ± 10.3	53.5 ± 9.4	*55.5 ± 10.2	52.4 ± 9.5	55.4 ± 10.1	51.9 ± 10.0
BMI (kg/m^2^)	23.3 ± 3.2	23.4 ± 3.6	23.3 ± 3.3	23.4 ± 3.5	23.3 ± 3.3	23.4 ± 3.7
KOA (%)	**33.8	55.0	**37.2	59.7	*40.0	42.4

*HV* hallux valgus, *BMI* body mass index, *KOA* knee osteoarthritis

**p < 0.01, *p < 0.05

**Fig. 2:**
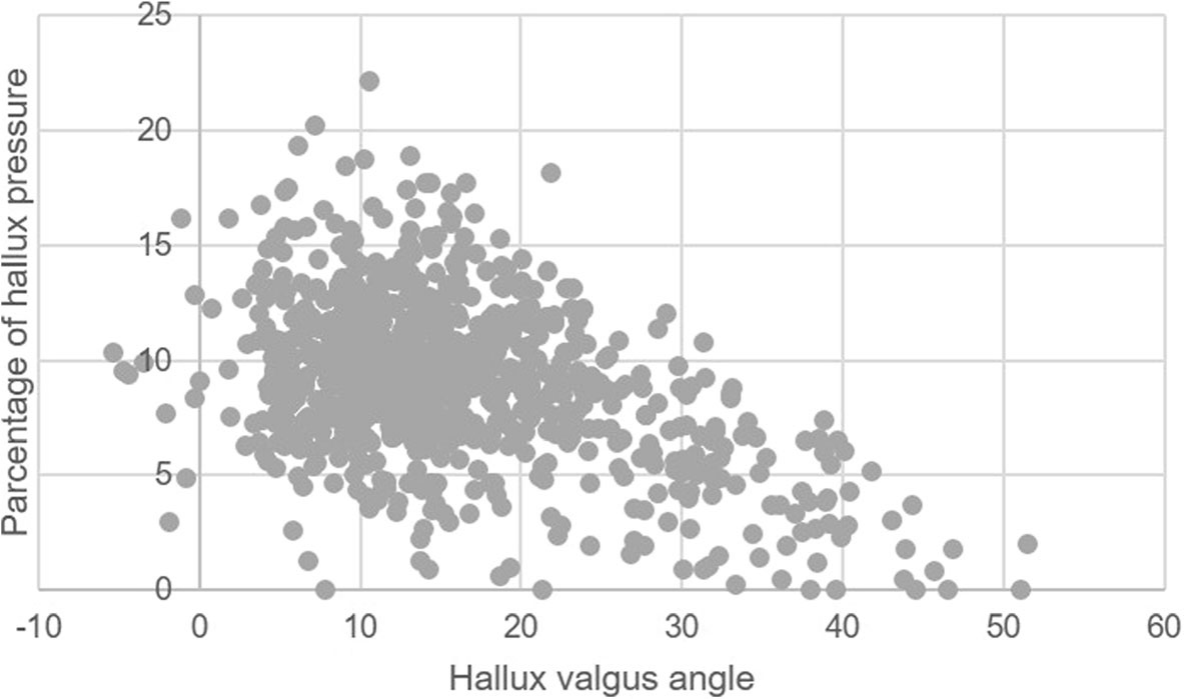
Relationship between the percentage of hallux footprint and the hallux valgus angle

**Fig. 3:**
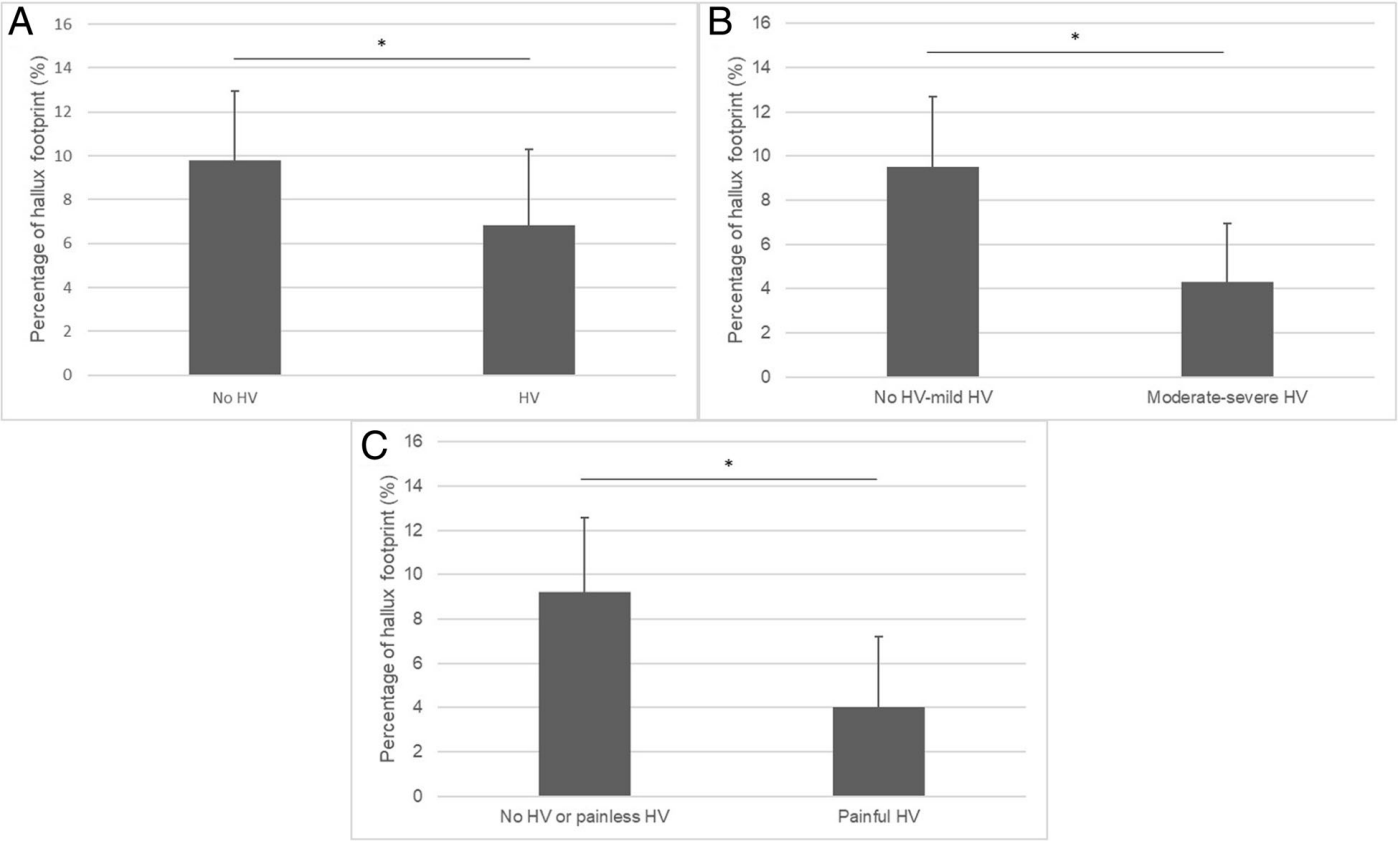
Comparison of the percentage of the hallux footprint between the no HV group and the HV group (**a**), between the no HV-mild HV group and the moderate-severe HV group (**b**), and between the no HV or painless HV group and the painful HV group (**c**). HV hallux valgus. *p < 0.01. Mann-Whitney test

The relationships between HV and physical functions are shown in Tables 2 and 3. Table 2 shows the results after adjustment for age, sex, and BMI, and Table 3 shows the results after adjustment for age, sex, BMI, and KOA. There was no significant difference between the HV group and the no HV group in physical functions. On the other hand, grip strength and maximal 6-m walking time were significantly stronger and faster in the no HV-mild HV group than in the moderate-severe HV group, respectively. In terms of HV-related pain, both usual and maximum walking speeds were significantly slower in the painful HV group than in the no HV or painless HV group. During 6-m walking, a significant negative correlation was observed between the HV angle and the percentage of hallux footprint (Fig. 2). Pearson’s correlation coefficient (r) and the corresponding *p* value were *r* = − 0.44 and *p* < 0.01. The percentage of hallux footprint was significantly higher in no HV feet than in HV feet (Fig. 3a). The percentage of hallux footprint was significantly higher in no HV or mild HV feet than in moderate-severe HV feet (Fig. 3b). Moreover, the percentage of hallux footprint was significantly higher in no HV or painless HV feet than in painful HV feet (Fig. 3c).

**Table 2:** Comparison of physical functions between the no HV group and the HV group, between the no HV-mild HV group and the moderate-severe HV group, and between the no HV or painless HV group and the painful HV group after adjusted for age, sex, and body mass index

		No HV	HV	Standard partial regression coefficient	*p* Value	No HV-mild HV	Moderate-severe HV	Standard partial regression coefficient	*p* Value	No HV or painless HV	Painful HV	Standard partial regression coefficient	*p* Value
		(*n* = 402)	(*n* = 160)			(*n* = 495)	(*n* = 67)			(*n* = 529)	(*n* = 33)		
Grip strength, kg	Crude	28.6 ± 8.5	25.1 ± 6.2			28.2 ± 8.2	23.1 ± 6.0			27.9 ± 8.1	23.2 ± 7.0		
	Adjusted	27.6 ± 5.2	27.6 ± 5.2	0.007	0.910	27.8 ± 5.1	26.3 ± 5.2	−0.140	*0.034	27.7 ± 5.1	26.4 ± 5.1	−0.088	0.189
Single-leg stance time, s	Crude	31.2 ± 22.8	24.8 ± 22.1			30.0 ± 22.7	24.3 ± 22.7			29.8 ± 22.7	22.5 ± 22.9		
	Adjusted	30.1 ± 18.3	27.4 ± 18.6	−0.079	0.125	29.6 ± 18.2	27.3 ± 18.5	− 0.051	0.332	29.5 ± 18.2	26.3 ± 18.3	− 0.052	0.323
Stand up from a chair time, s	Crude	10.1 ± 3.6	11.1 ± 4.5			10.2 ± 3.6	11.5 ± 5.5			10.3 ± 3.8	11.8 ± 4.3		
	Adjusted	10.3 ± 3.4	10.7 ± 3.5	0.063	0.175	10.3 ± 3.4	11.2 ± 3.5	0.089	0.056	10.3 ± 3.4	11.3 ± 3.4	0.077	0.106
Usual gait speed, m/s	Crude	1.01 ± 0.21	1.01 ± 0.24			1.02 ± 0.21	1.00 ± 0.26			1.02 ± 0.22	0.92 ± 0.25		
	Adjusted	1.01 ± 0.20	1.03 ± 0.20	0.039	0.376	1.02 ± 0.20	1.01 ± 0.20	−0.003	0.949	1.02 ± 0.21	0.93 ± 0.20	− 0.108	*0.017
Maximum gait speed, m/s	Crude	1.33 ± 0.27	1.28 ± 0.31			1.33 ± 0.27	1.22 ± 0.33			1.33 ± 0.27	1.17 ± 0.37		
	Adjusted	1.32 ± 0.24	1.32 ± 0.24	0.002	0.968	1.33 ± 0.24	1.26 ± 0.24	−0.103	*0.037	1.33 ± 0.23	1.22 ± 0.24	−0.120	*0.016

*HV* hallux valgus, *M* male, *F* female, *s* second, *m/s* meters per second

*p < 0.05

**Table 3:** Comparison of physical functions between the no HV group and the HV group, between the no HV-mild HV group and the moderate-severe HV group, and between the no HV or painless HV group and the painful HV group after adjustment for age, sex, body mass index, and knee osteoarthritis

		No HV	HV	Standard partial regression coefficient	*p* Value	No HV-mild HV	Moderate-severe HV	Standard partial regression coefficient	*p* Value	No HV or painless HV	Painful HV	Standard partial regression coefficient	*p* Value
		(*n* = 402)	(*n* = 160)			(*n* = 495)	(*n* = 67)			(*n* = 529)	(*n* = 33)		
Grip strength, kg	Crude	28.6 ± 8.5	25.1 ± 6.2			28.2 ± 8.2	23.1 ± 6.0			27.9 ± 8.1	23.2 ± 7.0		
	Adjusted	27.6 ± 5.2	27.7 ± 5.3	0.007	0.813	27.7 ± 5.1	26.4 ± 5.2	−0.055	*0.043	27.7 ± 5.1	26.5 ± 5.1	−0.035	0.206
Single-leg stance time, s	Crude	31.2 ± 22.8	24.8 ± 22.1			30.0 ± 22.7	24.3 ± 22.7			29.8 ± 22.7	22.5 ± 22.9		
	Adjusted	30.1 ± 18.4	27.6 ± 18.7	−0.049	0.162	29.6 ± 18.2	27.5 ± 18.6	− 0.03	0.391	29.5 ± 18.2	26.5 ± 18.3	−0.032	0.351
Stand up from a chair time, s	Crude	10.1 ± 3.6	11.1 ± 4.5			10.2 ± 3.6	11.5 ± 5.5			10.3 ± 3.8	11.3 ± 4.3		
	Adjusted	10.3 ± 3.5	10.7 ± 3.5	0.048	0.222	10.3 ± 3.4	11.1 ± 3.5	0.069	0.072	10.3 ± 3.4	11.8 ± 3.4	0.059	0.118
Usual gait speed, m/s	Crude	1.01 ± 0.21	1.01 ± 0.24			1.02 ± 0.21	1.00 ± 0.26			1.02 ± 0.22	0.92 ± 0.25		
	Adjusted	1.01 ± 0.20	1.03 ± 0.21	0.044	0.279	1.01 ± 0.20	1.02 ± 0.20	0.003	0.931	1.02 ± 0.20	0.94 ± 0.20	−0.091	*0.021
Maximum gait speed, m/s	Crude	1.33 ± 0.27	1.28 ± 0.31			1.33 ± 0.27	1.22 ± 0.33			1.33 ± 0.27	1.17 ± 0.37		
	Adjusted	1.32 ± 0.24	1.32 ± 0.24	0.007	0.849	1.33 ± 0.24	1.27 ± 0.24	−0.072	*0.048	1.33 ± 0.23	1.23 ± 0.24	−0.084	*0.018

*HV* hallux valgus, *M* male, *F* female, *s* second, *m/s* meters per second

*p < 0.05

## Discussion

The results of this cross-sectional study indicated that participants with moderate-severe HV or painful HV had impaired physical functions compared to participants with no HV-mild HV or painless HV after taking into account age, sex, BMI, and radiographic KOA. Hallux loading was reduced with increasing HV severity or HV-related pain.

With regard to standing balance, Menz et al. [21], Spink et al. [22], and Nix et al. [11] reported that poorer lateral stability, poorer coordinate stability, and increased postural sway were associated with HV in elderly populations. On the other hand, Mickle et al. [23] and Menz and Lord [24] showed no relationship between HV and standing balance. These previous reports included much finer analyses, with evaluation of the center of pressure (COP) using a force plate, and/or more severe conditions, such as eyes closed, than the present study. The data of the present study were only for standing time with eyes open to examine standing balance, but there was no relationship between HV and standing balance. If finer evaluations, such as analyzing COP, and/or more severe conditions, such as eyes closed, were performed, some differences might be detected.

To the best of our knowledge, there have been no reports of the relationship between HV and hand grip strength. The present study showed that moderate-severe HV deformity (HV angle ≥30°) was associated with impaired hand grip strength. It is easy to imagine that patients with moderate-severe HV have smaller grip strength of the foot because of poor alignment of the 1st MTP joint. Grip strength of the hallux might be correlated with hand grip strength. Thus, patients with poor grip strength in the moderate-severe HV group may show poor grip strength of the hallux. However, this is just a hypothesis, and further studies are needed.

No significant between-group differences were found in walking performance in some studies [11, 22]. The present data for the no HV group and the HV group are consistent with these previous studies. However, the definitions used in the various studies differed, since the data were self-reported (such as the Manchester scale) or HV < 20° was used, so that the HV groups had many mild HV participants. In the present study, maximum walking speed was faster in the no HV-mild HV group than in the moderate-severe HV group. Cho et al. [4] showed that participants with moderate or greater HV (HV angle > 25°) had significantly worse functional status, foot health function status, and self-assessment of their foot condition. These data support the present data. There were no data about maximum walking speed in previous studies, but maximum walking requires a higher level of physical function than usual walking. Thus, moderate-severe HV appeared to be associated with maximum walking speed in the present study.

Previous studies reported that foot pain was associated with poor physical function. Some studies [24–26] reported that participants with foot pain had more difficulty walking than participants without foot pain. In addition, some reports [1, 4] showed that HV was associated with more self-reported foot pain and poorer self-reported physical function. Abhishek et al. [10] further highlighted the importance of hallux pain with HV, reporting that health-related quality of life was progressively impaired in participants with HV alone, hallux pain alone, and HV with hallux pain. In the present study, walking speeds of participants with painful HV were slower than those of participants with no HV or mild HV.

Previous reports [27–29] showed an inverse relationship between HV severity and reduced hallux loading. Nix et al. [11] and Mickle et al. [13] showed that participants with HV had decreased hallux plantar flexion strength. Sanders et al. [30] reported an inverse relationship between HV angle and hallux plantar flexion strength, as well as lower mean hallux plantar flexion strength in those with painful HV than in those with HV without complaints. Moreover, Mickle et al. [23] reported less hallux loading in HV patients than controls. Hurn et al. [29] reported that participants with moderate (average HV angle = 30.8°) and severe HV (average HV angle = 39.9°) showed significantly reduced hallux plantar pressure-time compared to controls. These reports are consistent with the results of the present study. These reduced hallux plantar pressures might reflect impaired physical function, such as slower walking speed.

Caution must be applied when comparing reports from different studies [1]. Some studies have used self-report information, such as the Manchester scale [21–23, 31], and others have used X-ray definitions. In terms of X-ray examinations, studies have used a range of definitions, such as HV angle > 15° or 20° and so on. The present study used standardized weight-bearing radiographs, and the definition of HV was an HV angle > 20° according to the definition of the Japanese Foot and Ankle Society.

The present study has several limitations. First, since it was a cross-sectional study, causal relationships cannot be determined. Second, participants could walk to the survey site and could understand and sign an informed consent form. Thus, elderly participants in this study were relatively healthier and had no dementia. Furthermore, since workers in Japan usually retire between 63 and 65 years old, male participants in their 50s usually have health examinations at their work place. Thus, male participants in their 50s who worried about their health tended to attend our exam. Due to this healthy user bias, the participants in this study do not truly represent the general population. The percentage of men in this study was 34.5%, but the percentage of men over 65 years old in this village was 39.2% in 2009. Further, the percentage of men over 65 years old in Japan was 42.8% in 2009. This sex mismatch is also one of the limitations. Third, the examinations were held in a specific mountain village, so that this study is not necessarily representative of all of Japan. Fourth, the balance test was simply single-leg standing time with eyes open. If a standing test with eyes closed and/or postural sway using a force plate were used, significant differences might be detected in the balance tests. Fifth, hallux pain is not always due to HV. Thus, painful HV does not necessarily mean pain related to HV.

## Conclusions

This cross-sectional epidemiological study showed that moderate-severe HV impaired some physical functions (grip strength and maximum walking speed), and painful HV slowed walking speed regardless of radiographic KOA. Hallux plantar pressure decreased according to HV angle and pain. We suspect this reduced hallux pressure is one of the reasons for the functional impairments in participants with moderate-severe HV or painful HV. By controlling pain and treating severe HV deformity with treatments such as surgery, the physical functions of HV patients might be improved.

## Abbreviations

BMI: body mass index
HV: hallux valgus
KOA: knee osteoarthritis
